# Inborn Errors of Immunity in Adults with Autoimmune Liver Diseases

**DOI:** 10.5152/tjg.2024.23171

**Published:** 2024-07-01

**Authors:** Şefika Nur Ayar, Elif Soyak Aytekin, Cem Şimşek, Osman Dağ, Deniz Çağdaş, Yasemin H. Balaban

**Affiliations:** 1 Department of Internal Medicine, Hacettepe University Faculty of Medicine, Ankara, Türkiye; 2 Department of Pediatric Immunology, Hacettepe University İhsan Doğramacı Children’s Hospital, Ankara, Türkiye; 3 Division of Gastroenterology, Department of Internal Medicine, Hacettepe University Faculty of Medicine, Ankara, Türkiye; 4 Department of Biostatistics, Hacettepe University Faculty of Medicine, Ankara, Türkiye

**Keywords:** Inborn errors of immunity, autoimmunity, autoimmune liver diseases, autoimmune hepatitis, primary biliary cholangitis, primary sclerosing cholangitis

## Abstract

**Background/Aims:**

Inborn errors of immunity (IEI) may associate with autoimmune diseases, including autoimmune liver diseases (AILD). However, both the IEI frequency and secondary effects of immunosuppressives are unknown in patients with AILD due to the lack of data. We aimed to evaluate the ratio of IEI in AILD.

**Materials and Methods:**

A total of 82 patients with AILD (39 autoimmune hepatitis, 32 primary biliary cholangitis, 7 variant syndromes (VS), and 4 primary sclerosing cholangitis patients) were included in this single-center, cross-sectional, and descriptive study. The patients were evaluated and classified according to diagnostic criteria for IEI.

**Results:**

Out of 82 patients with AILD, female/male ratio was 3.6. Median age of diagnosis of AILD was 45 years. We diagnosed 15 (18%) patients with immunodeficiency (ID). Inborn errors of immunity ratio was highest in VS patient group (29%). Out of 15 patients with ID, 4 (4.8%) patients had common variable immunodeficiency, 4 (4.8%) had partial immunoglobulin A deficiency, 4 (4.8%) had selective immunoglobulin M deficiency, and 3 (3.6%) had combined immunodeficiency.

**Conclusion:**

We detect ID in about one-fifth of the patients with AILD. The present study showed a significant risk of IEI that is blurred by the shadow of immune suppressive treatments. We suggest that the AILD patients with ID will benefit from the individualized and targeted therapeutic options used in IEI. Further research with larger patient groups and long-term follow-up are desperately needed to elucidate the diagnostic, therapeutic, and prognostic impacts of IEI-related individualized therapy on AILD patients.

**Main Points**

- In this study, immunodeficiency was detected in 18% (15/82) of patients with autoimmune liver diseases.
- Among autoimmune liver disease patients, lower and upper respiratory tract infections were more frequent in patients who were diagnosed with immunodeficiency.
- Detection of coexisting/underlying primary immunodeficiency in autoimmune liver patients may help predict the prognosis of the disease and pave the way for the development of personalized treatments.

## Introduction

As the liver drains blood from both caval and portal systems, it is exposed to a variety of antigens from the diet, intestinal microbiota, and autoantigens. With such an extensive antigenic stimulus and without a generalized immunologic response, the liver can be considered as an “immune tolerogenic” organ rather than an “immune reactive” one.^1^ The inherent immune mechanisms of liver balance surveillance with tolerance. Breakdown of the balance leads to the development of a spectrum of autoimmune liver diseases (AILD); autoimmune hepatitis (AIH), primary biliary cholangitis (PBC), primary sclerosing cholangitis (PSC), and variant syndrome (VS). Our knowledge regarding the pathogenesis of loss of self-tolerance in the liver is limited to some molecular and cellular mechanisms involving dysfunctions in cells, including T regulatory and Kupffer cells, and altered gut–liver axis, which remains to be elucidated in more detail.^2^

Inborn errors of immunity (IEI) are rare and heterogeneous group of genetic disorders that affect the development and/or function of the immune system.^3^ More than 450 genes that cause IEI have been identified until now. Clinical presentations of IEI are not limited to infections but also include allergy, lymphoproliferation, autoinflammation, autoimmunity, and other manifestations due to immune dysregulation. The rate of autoimmune diseases in IEI is reported to be 26.2%.^4^ Along all other autoimmune diseases, AILD also develops at a high frequency in patients with IEI.^5-7^ However, the prevalence of IEI in patients with AILD has not been investigated due to the difficulties in studying the coexistence of these 2 rare and heterogenous group of diseases.

The patients with AILD are referred to the gastroenterology clinics. Generally, the therapy, mostly immunosuppressive medication, is given without routine immunologic evaluation. Consequently, the altered immune parameters in these patients are mostly attributed to the effects of immunosuppressives.

Our hypothesis is that IEI may be an underlying factor in a definite ratio in patients with AILD, and the frequency of IEI might be higher in AILD patients than the general population. We aimed to test our hypothesis through immunological evaluation of patients with AILD. Elucidating the association of AILD and IEI will pave the way for a better understanding of AILD pathophysiology and the definition of individualized therapeutic options.

## Materials and Methods

We conducted this single-center, cross-sectional, and observational study at Hacettepe University Faculty of Medicine Hospital, Gastroenterology unit, and Pediatric Immunology units between January and July 2020. We included the patients older than 18 years of age who met current AIH, PBC, PSC, or VS criteria.^8-11^ The patients were under stable doses of drug treatment for the last 3 months and they approved the informed consent. For AIH criteria, we accepted “The International Autoimmune Hepatitis Group” score of at least 12.^8^ The International Autoimmune Hepatitis Group criterion is a scoring system that includes categories of gender, alkaline phosphatase/alanine aminotransferase (ALP/ALT) ratio, immunoglobulin G (IgG) level, autoantibody titers, viral hepatitis marker negativity, presence of concomitant autoimmunity, alcohol use, hepatotoxic drug use, HLA DR3 or DR4 positivity, liver histology, and treatment response. For PBC and PSC criteria, we used the current American Association for the Study of Liver Diseases guidelines.^9,10^ A diagnosis of PBC was established if at least 2 of the following are present; ALP 1.5 times or more upper limit of normal (ULN), presence of antimitochondrial antibody (AMA) at a titer of 1 : 40 or higher, histologic evidence of PBC. Primary sclerosing cholangitis diagnosis was made based on the presence of the cholestatic pattern of liver test abnormalities (especially ALP elevation), cholangiographic evidence of characteristic bile duct changes, and by excluding secondary causes of sclerosing cholangitis. We used the Paris criteria for AIH and PBC variant/overlap syndrome.^11^ The Paris criteria for the AIH-PBC variant requires 2 of 3 features associated with PBC selected from the following: serum ALP level ≥2-fold ULN or γ-glutamyl transferase (GGT) level ≥5-fold ULN, AMA positivity, and compatible histology; They also require interface hepatitis associated with AIH and on of the following: serum alanine aminotransferase level ≥5-fold ULN, IgG level ≥2-fold ULN, or the presence of smooth muscle antibodies. Since there was no international guideline for diagnosing the AIH and PSC variant, we made the clinical diagnosis in patients with clinical and laboratory features of both AIH and PSC. Exclusion criteria were the presence of concomitant liver diseases, acute decompensation of liver disease, active chemotherapy, or hematopoietic stem cell transplantation.

We interviewed with each patient by using the IEI screening questionnaire of the Jeffrey Modell Foundation.^12^ We also obtained a detailed history, including past infections, comorbidities, and family history. The laboratory results dating before the initiation of immunosuppressive therapy for AILD were collected from electronic records includes complete blood count, ALT, aspartate aminotransferase, ALP, GGT, direct and indirect bilirubin, albumin, prothrombin time and international normalized ratio, activated partial thromboplastin time, creatinine, urea, hepatitis B surface antigen, hepatitis B surface antibody, hepatitis B core antibodies (anti-HBc IgG and IgM), hepatitis C antibody, anti-human immunodeficiency virus, anti-hepatitis A virus antibody, antinuclear antibody (ANA), AMA, anti-smooth muscle antibody (ASMA), liver kidney microsomal antibody type 1 (LKM-1), IgG, immunoglobulin M (IgM), immunoglobulin A (IgA), immunoglobulin E, complement 3 and 4, hepatobiliary ultrasound, and upper abdomen magnetic resonance imaging.

The patients were further analyzed in the pediatric immunology department after consultation. Further immunological tests, antibody responses, lymphocyte subsets, T-B lymphocyte subgroups were done prospectively during the study period while the patients were under therapy. Some of the abnormal immune-related parameters of the patients were before, but some were after the immunosuppressive therapy. We assumed as if they have a primary immunodeficiency (ID) at first evaluation.

Inborn errors of immunity are diagnosed with the diagnostic criteria defined for each of the diseases.^13^ In the follow-up period, none of the immune parameters compatible with the primary ID diagnosis resolved. We grouped all patients as patients with and without ID.

Ethics approval was obtained from the Ethics Committee of Hacettepe University for this study (study approval identification code: GO 19/1122, Date: December 3, 2019).

### Statistical Analysis

The steps of data analysis were implemented using Statistical Package for the Social Sciences Statistics software, version 22.0 (IBM Corp., Armonk, NY, USA) and R programming language. While analyzing quantitative variables, normality of data in each group was tested with Shapiro–Wilk test. Mann–Whitney *U*-test was conducted to compare 2 groups and median (minimum–maximum) was reported to describe the variables since the normality assumption is not met. Pearson chi-square test, Pearson chi-square test with Yates correction, and Fisher’s exact test were utilized to investigate the relation between categorical variables. Frequency and percentage were reported to describe categorical variables.

The figures were drawn using ggplot2 R package. The packages readxl, janitor, and onewaytests were used for data import, data manipulation, and one-way analysis parts in R.

## Results

### Demographic and Clinical Findings

Out of 111 patients with AILD, 89 patients were included in the study; 9 patients did not give informed consent, 6 patients’ diagnoses did not meet the current diagnostic criteria of any of the AILD, 3 patients had acute liver decompensation, 2 patients were on active chemotherapy, 1 patient had accompanying Wilson disease, 1 patient underwent hematopoietic stem cell transplantation due to lymphoma. Evaluation for IEI could not be completed in 7 patients (4 AIH, 2 PSC, and 1 VS) due to coronavirus disease 2019 pandemic. Therefore, remaining 82 patients were included in the final analysis: 39 AIH, 30 PBC, 6 PSC, and 7 VS.

**Figure 1.**
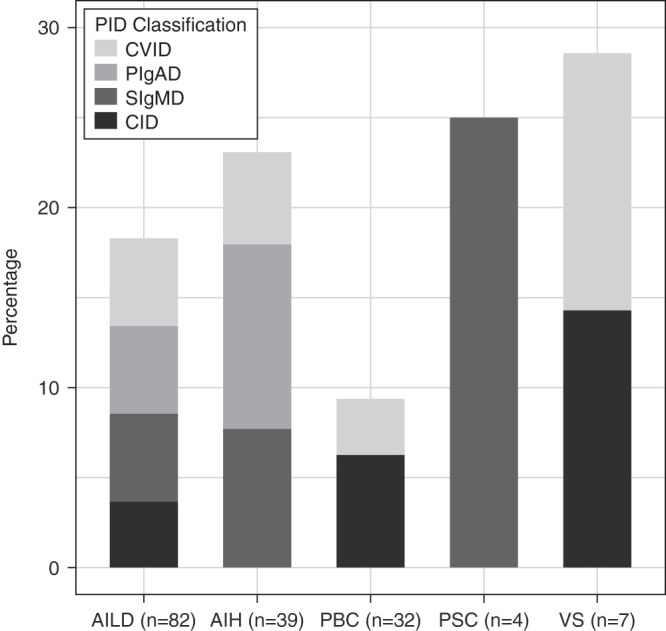
Frequency and distribution of immunodeficiency in AILD groups. Immunodeficiency was detected in 18% (15/82) of AILD patients (the number of diseases are given according to IEI classification). Immunodeficiencies were detected in 23% of AIH (9/39 patients; 4 PIgAD, 3 SIgMD, and 2 CVID), 9% of PBC (3/32 patients; 2 CID and 1 CVID), 25% of PSC (1/4 patient; 1 SIgMD), and 29% of VS (2/7 patients; 1 CID and 1 CVID). AIH, autoimmune hepatitis; AILD, autoimmune liver disease; CID, combined immunodeficiency; CVID, common variable immunodeficiency; IEI, inborn errors of immunity; PBC, primary biliary cholangitis; pIgAD, partial IgA deficiency; PSC, primary sclerosing cholangitis; SIgMD, selective immunoglobulin M deficiency; VS, variant syndrome.

Immunodeficiency was detected in 18% (15 patients) of patients with AILD (Figure 1). Four (4.8%) patients were diagnosed with common variable ID (CVID), 4 (4.8%) patients with partial IgA deficiency (pIgAD), 4 (4.8%) patients with selective IgM deficiency (sIgMD), and 3 (3.6%) patients with combined ID (CID).

Immunodeficiency diseases were detected in 29% of VS (2/7 patients; 1 CID and 1 CVID); 25% of PSC (1/4 patient; 1 sIgMD); 23% of AIH (9/39 patients; 4 pIgAD, 3 sIgMD, and 2 CVID) and 9% of PBC (3/32 patients; 2 CID and 1 CVID). Four patients with CVID were previously diagnosed.

The demographic and clinical features and laboratory results of AILD patients with and without ID are summarized in Table 1 and Table 2, respectively. All laboratory values shown in Table 2 belong to time before the initiation of any treatment for AILD disease. There were no significant differences between groups for age, gender, age at diagnosis of AILD, and presence of cirrhosis (*P* > .05).

Patients with ID were younger (37 vs. 49 years of age) (*P* = .054). The frequencies of autoimmunity or malignancy accompanying AILD, familial history of autoimmunity or malignancy, and parental consanguinity were also similar among patients with and without ID (*P* > .05). While thrombocyte counts were lower in patients with ID than those without ID (217 vs. 260/mL, *P* = .013), thrombocytopenia rate was similar (20% vs. 10%, *P* = .36). Patients with ID had lower GGT (68 vs. 115 IU/L, *P* = .047). Patients with ID had lower serum albumin levels (4.1 vs. 4.4 mg/dL, *P* = .056). As expected, seronegativity for autoimmune markers (namely ANA, ASMA, LKM-1, or AMA) was higher in patients with ID and AIH (22% (2/9) vs. 3% (1/30), *P* = .127) and with ID and PBC (67% (2/3) vs. 14% (4/29), *P* = .083). Complete blood count and serum immunoglobulin levels of patients with ID are given in detail in Supplementary Table 1.

**Figure 2.**
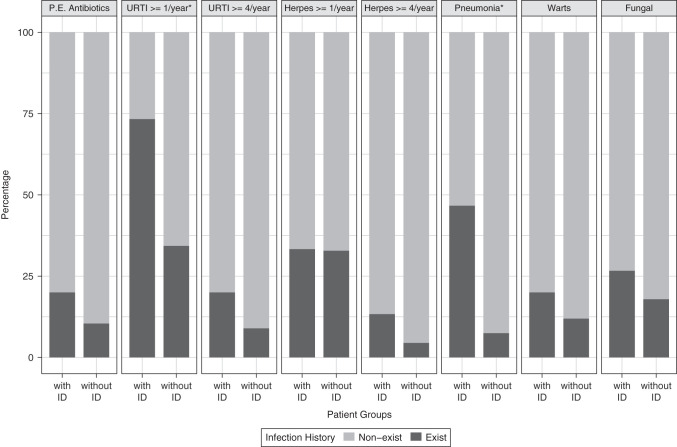
Infection history in AILD patients with and without immunodeficiency. Comparison percentages of patients with and without ID in terms of infection history. Those marked with an asterisk are statistically significant (*P* < .05). AILD, autoimmune liver disease; ID, immunodeficiency; URTI, upper respiratory tract infection; P.E., parenteral.

The annual rate of upper respiratory tract infections was significantly higher in patients with ID (73% vs. 34%, *P* = .013) (Figure 2). The history of frequent upper respiratory tract infections (≥4 times in a year) was 20% in ID patients, while only 9% in those without ID (*P* = .013). Additionally, the annual rate for pneumonia was significantly higher in ID patients (48% vs. 7%, *P* = .001). On the other hand, infectious disease rates for herpes, warts, and fungal infection were similar in patients with and without ID (*P* > .05) (Supplementary Table 2).

### Follow-Up and Therapy

The median follow-up of AILD patients was 7.5 (range 0-31) years. Eighteen (46%) patients with AIH were under steroid and azathioprine combination, 12 (31%) steroids, 6 (15%) azathioprine, and 3 (8%) mycophenolate mofetil. All PBC and PSC patients were using ursodeoxycholic acid (UDCA). Four patients with VS were using steroids and UDCA, 1 using steroids alone, and 1 tacrolimus and mycophenolate mofetil combination after liver transplantation. The immunosuppressive treatments used by patients with and without ID for AILD are shown in the table (Table 3).

All 4 patients who were diagnosed with CVID and 1 patient with CID are currently under the treatment of monthly intravenous immunoglobulin, and their liver diseases are in remission. Other newly diagnosed patients with ID patients have been following up without any immunotherapy. In this study, we couldn’t find any significant difference in AILD treatment responses of patients with and without ID (*P* > .05) (Table 2).

## Discussion

Although the association of autoimmunity with IEI is well known, the frequency of IEI among AILD patients has not been defined. In this retrospective, single-center cohort study, ID was present in 15 (18%) of 82 patients with AILD who fulfilled the diagnostic criteria for IEI. The prevalence of IEI in the general population is estimated to be 1 : 10 000 but may be more frequent in populations with a high consanguineous marriage ratio.^14^ As such, it is estimated that IEI prevalence is relatively higher in Türkiye, where the ratio of consanguineous marriages is 24% in 2018.^15-17^ In this study, we diagnosed 18% (15/82) of AILD patients with ID. If all the ID diagnoses were inborn/primary, the frequency will be significantly higher than estimations for the general population.

Previous data regarding the association of AILD with ID is limited to studies that investigate the frequency of autoimmune diseases in patient cohorts with IEI. In different IEI syndromes, AIH was reported to range between 1.6% and 43.0%.^18-22^ Primary biliary cholangitis was detected in 1.2% of patients with CVID.^23^ Primary sclerosing cholangitis was reported to accompany various types of IEI in children^24^ and was present as high as 45% in patients with hyper IgM syndrome.^25^

The diagnosis of a concomitant IEI and AILD is challenging, because, immunosuppressive medication may alter some of the immunological parameters, such as autoantibody titers and Ig levels. Immunological parameters that decrease under immunosuppression will normalize in some of the patients after a while. There are several case reports emphasizing the difficulties in diagnosing AIH with standard diagnostic criteria in CVID patients who have low immunoglobulin levels.^26-28^ Furthermore, Fukushima et al^26^ suggested that some CVID cases reported as “non-B non-C hepatitis” who benefited from immunosuppressive therapy might have AIH. Similarly, some cases were described in the literature as “chronic hepatitis” accompanying CVID^29^ and those may be undiagnosed AIH patients with accompanying IEI. In the present study, seronegativity rates among AIH and PBC patients were higher in the ID group than in the group without ID though the difference remained statistically insignificant.

Lower and upper respiratory tract infections were more frequent in patients who were diagnosed with ID in this study. Although AILD treatment responses of patients with and without ID were similar in this study, high infection rates in ID patients may have a potential risk for the course of AILD. Furthermore, some of the patients with IEI may need hematopoietic stem cell transplantation in addition to liver transplantation, which will increase morbidity and mortality.

We suggest that AILD patients with a history of frequent, severe, or opportunistic infections, additional autoimmune, inflammatory, allergic, lymphoproliferative diseases, or malignancies should be evaluated for the associated IEI.^30^ The first step laboratory parameters for immunologic evaluation will be the complete blood count and immunoglobulin levels, which are generally done in the diagnosis of AILD, Suspected patients of IEI should be consulted as the wait and see approach and the diagnostic delay will increase morbidity and mortality.

To the best of our knowledge, this study is the first study that investigates the frequency of ID in AILD. The significantly higher frequency of ID among AILD than the general population and the lack of awareness about investigating IEI in AILD has been revealed. However, there are some limitations. First, this is a single-center study, and the number of patients, especially PSC which has well-defined association with IEI was limited (only 4 patients).^24^ Second, the impact of ID on the diagnosis and prognosis of AILD could not be defined as the study does not include long-term follow-up. Lastly, genetic analysis was not yet performed as the patients are still under immunosuppressive therapy. Further research with larger multicenter patient series is certainly needed to evaluate the prevalence of IEI and their clinical impacts on AILD diagnosis, treatment response, and prognosis.

The present study revealed that it is not only possible to better diagnose and classify patients with AILD, but also improve the treatment outcomes with the help of immunological evaluation. We suggest that the patients with AILD will benefit from the individualized and targeted therapeutic options used frequently in immunology.

## Figures and Tables

**Table 1.** Demographic and Clinical Features of Patients with Autoimmune Liver Disease

	All Patients (n = 82)	With ID (n = 15)	Without ID (n = 67)	P*
Female, n (%)	64 (78)	11 (73)	53 (79)	.980^b^
Median age, years (minimum–maximum)	49 (18-75)	37 (19-60)	49 (18-75)	.054^c^
Median age at diagnosis of AILD, years (minimum–maximum)	45 (6-72)	34 (9-60)	45 (6-72)	.128^c^
AILD, n (%) AIH PBC PSC VS	39 (48) 32 (39) 4 (5) 7 (9)	9 (60) 3 (20) 1 (7) 2 (13)	30 (45) 29 (43) 3 (5) 5 (7)	.405^a^
Extrahepatic autoimmunity, n (%)	28 (34)	5 (33)	23 (34)	1.000^d^
Malignancy, n (%)	7 (9)	1 (7)	6 (9)	1.000^b^
Parental consanguinity, n (%)	23 (28)	4 (27)	19 (28)	1.000^b^
Autoimmunity in family, n (%)	26 (31)	7 (47)	19 (28)	.221^b^
Malignancy in family, n (%)	27 (33)	6 (40)	21 (31)	.552^b^
Cirrhosis, n (%)	10 (12)	2 (13)	8 (12)	1.000^b^
Hepatomegaly, n (%)	18 (21)	5 (33)	13 (21)	.335^b^
Splenomegaly, n (%)	21(26)	7 (46)	14 (23)	.102^b^
Treatment response, n (%) New diagnoses Sufficient Insufficient	6 (7) 68 (83) 8 (10)	1 (7) 11 (73) 3 (20)	5 (7) 57 (85) 5 (7)	.762^a^

Results are reported as median (minimum–maximum) for the continuous variables not satisfying the normality assumption. Categorical variables are reported as frequency and percentages.

AIH, autoimmune hepatitis; AILD, autoimmune liver disease; n, sample size; PBC, primary biliary cholangitis; ID, immunodeficiency; PSC, primary sclerosing cholangitis; VS, variant syndrome.

*Statistical analysis is done between with and without ID groups.

^a^Pearson’s chi-square test.

^b^Fisher’s exact test.

^c^Mann–Whitney *U*-test.

^d^Pearson’s chi-square test with Yates correction.

**Table 2.** Initial Laboratory Parameters of Patients with Autoimmune Liver Disease

	All Patients (n = 71)	With ID (n = 14)	Without ID (n = 57)	P*
Hemoglobin (g/dL) (minimum–maximum)	13.0 (8.1-17.0)	13.5 (9.4-16.7)	12.9 (8.1-17.0)	.278^d^
Leukocyte (/mL) (minimum–maximum)	6.9 (3.5- 14.3)	6.0 (4.1-10.8)	6.9 (3.5-14.3)	.552^d^
Neutropenia (<1.5 × 10^3^/mL), n (%)	2 (3)	1 (7)	1 (2)	.358^b^
Lymphopenia (<1.3 × 103/mL), n (%)	7 (9)	1 (7)	6 (11)	.000^b^
Thrombocyte (/mL) (minimum–maximum)	247 (73-494)	217 (84-286)	260 (73-494)	.013^d^
AST (IU/L) (minimum–maximum)	64 (18-11179)	69 (26-409)	64 (18-11161)	.768^d^
ALT (IU/L) (minimum–maximum)	74 (12-2126)	74 (12-2126)	111 (25-437)	.501^d^
GGT (IU/L) (minimum–maximum)	108 (18-606)	68 (18-325)	115 (18-606)	.047^d^
ALP (IU/L) (minimum–maximum)	165 (62-2014)	167 (75-410)	163 (62-2014)	.334^d^
Albumin (mg/dL) (minimum–maximum)	4.2 (2.4-4.9)	4.4 (3.6-4.7)	4.1 (2.4-4.9)	.056^d^
T. Bilirubin (mg/dL) (minimum–maximum)	0.6 (0.2-24)	0.7 (0.3-2.4)	0.6 (0.2-24)	.783^d^
INR (minimum–maximum)	0.9 (0.7-2.1)	0.9 (0.7-2.1)	1 (0.8-1.4)	.543^d^
Creatinine (mg/dL) (minimum–maximum)	0.6 (0.2-0.9)	0.5 (0.2-0.9)	0.6 (0.2-0.9)	.194^d^

Results are reported as median (minimum–maximum) for the continuous variables not satisfying the normality assumption. Categorical variables are reported as frequency and percentages.

AILD, autoimmune liver disease; ALP, alkaline phosphatase; ALT, alanine aminotransferase; AST, aspartate aminotransferase; GGT, gamma-glutamyl transferase; INR, international normalized ratio; n, sample size; ID, immunodeficiency; VS, variant syndrome.

*Statistical analysis is done between with and without ID groups.

^a^Pearson chi-square test with Yates correction.

^b^Fisher’s exact test.

^c^Pearson chi-square test.

^d^Mann–Whitney *U*-test.

**Table 3.** Immunosuppressive Treatments Used by Patients With and Without Immunodeficiency for Autoimmune Liver Disease

	With ID	Without ID	*P*
Treatment for AIH			
Corticosteroids	4 (2 CVID, PIgAD, SIgMD)	8	.416^a^
Azathioprine	2 (PIgAD)	4	.607^a^
Corticosteroids +Azathioprine	2 (PIgAD,SIgMD)	16	.139^a^
Mycophenolate mofetil	1 (SIgMD)	2	.556^a^
Treatment for VS			
Corticosteroids	1 (CVID)	0	.333^a^
Corticosteroids +Azathioprine	0	4	.067^a^
Mycophenolate mofetil	1 (CID)	0	.333^a^

AIH, autoimmune hepatitis; AILD, autoimmune liver disease; CID, combined immunodeficiency; CVID, common variable immunodeficiency; ID, immunodeficiency; PIgAD, partial immunoglobulin A deficiency; SIgMD, selective immunoglobulin M deficiency; VS, variant syndrome.

^a^Fisher’s exact test.

**Supplementary Table 1.** Initial Complete Blood Count and Serum Immunoglobulin Levels of Autoimmune Liver Disease Patients with Immunodeficiency

AILD		IEI disease	Hemoglobin (g/dl)	Leukocyte (/ml)	Neutrophil (/ml)	Lymphocyte (/ml)	Thrombocyte (/ml)	IgG (mg/dl)	IgM (mg/dl)	IgA (mg/dl)
AIH	P1	CVID	9	4.3	1.2	2.8	91	464	38	<6.6
	P2	CVID	13	6.9	5.0	1.4	104	618	79	46
	P3	PIgAD	12	7.8	4.0	3.0	211	1440	134	82
	P4	PIgAD	13	10.8	7.6	2.0	152	2240	119	78
	P5	PIgAD	11	8.3	5.6	1.9	215	3080	233	58
	P6	PIgAD	15	5.6	3.0	2.0	219	1090	95	94
	P7	SIgMD	15	7.8	4.6	2.7	286	1010	44	243
	P8	SIgMD	17	10.8	7.2	2.6	228	2760	48	240
	P9	SIgMD	14	4.3	2.2	1.7	154	1170	45	267
PBC	P1	CID	14	5.4	3.9	0.9	273	887	48	135
	P2	CID	12	5.7	3.4	1.7	227	1040	263	211
	P3	CVID	13	4.1	2.4	1.4	84	764	11	<6.6
PSC	P1	SIgMD	16	5.3	2.7	2.1	247	1550	77	158
VS	P1	CVID	14	8.7	5.5	2.3	226	570	53	14
	P2*	CID	-	-	-	-	-	-	-	-

*This patient was diagnosed in another center, thus his inital data could not be accessed. Normal ranges of serum immunoglobulin levels for adult: IgG, 913-1884 mg/dl; IgM, 88-322 mg/dl: IgA, 139-378 mg/dl. AIH, autoimmune hepatitis; AILD, autoimmune liver disease; CID, combined immunodeficiency; CVID, common variable immunodeficiency; ID, immunodeficiency; P, patient; PBC, primary biliary cholangitis; pIgAD, partial IgA deficiency; PSC, primary sclerosing cholangitis; SIgMD, selective IgM deficiency; VS, variant syndrome.

**Supplementary Table 2.** History of Infections in Autoimmune Liver Disease Patients According to the Presence of Immunodeficiencies

	With documented ID (n = 15)	Without documented ID (n = 67)	*P*
Parenteral antibiotic use n (%)	3 (20)	7 (10)	.380^a^
Upper respiratory tract infection, n (%) ≥1 time(s) in a year ≥4 times in a year	11 (73) 3 (20)	23 (34) 6 (9)	.013^b^ .355^a^
Labial herpes, n (%) ≥1 time(s) in a year ≥4 times in a year	5 (33) 2 (13)	22 (33) 3 (5)	1.000^a^ ,225^a^
Pneumonia (≥1 time(s) in life), n (%)	7 (47)	5 (7)	.001^a^
Warts (≥1 time(s) in life), n (%)	3 (20)	8 (12)	.414^a^
Fungal infection (≥1 time(s) in life) n (%)	4 (27)	12 (18)	.477^a^

Categorical variables are reported as frequency and percentages. AILD, autoimmune liver disease; ID, immunodeficiency.

^a^Fisher’s exact test ^b^Pearson chi-square test with Yates correction
